# Direct and heterologous approaches to identify the LET-756/FGF interactome

**DOI:** 10.1186/1471-2164-7-105

**Published:** 2006-05-03

**Authors:** Cornel Popovici, Yael Berda, Fabien Conchonaud, Aurélie Harbis, Daniel Birnbaum, Régine Roubin

**Affiliations:** 1 Institut de Cancérologie de Marseille, Laboratoire d'Oncologie Moléculaire, Institut Paoli-Calmettes et UMR599 INSERM, 27 Bd. Leï Roure, 13009 Marseille, France

## Abstract

**Background:**

Fibroblast growth factors (FGFs) are multifunctional proteins that play important roles in cell communication, proliferation and differentiation. However, many aspects of their activities are not well defined. LET-756, one of the two *C. elegans *FGFs, is expressed throughout development and is essential for worm development. It is both expressed in the nucleus and secreted.

**Results:**

To identify nuclear factors associated with LET-756, we used three approaches. First, we screened a two-hybrid cDNA library derived from mixed stages worms and from a normalized library, using LET-756 as bait. This direct approach allowed the identification of several binding partners that play various roles in the nucleus/nucleolus, such as PAL-1, a transcription regulator, or RPS-16, a component of the small ribosomal subunit. The interactions were validated by co-immunoprecipitation and determination of their site of occurrence in mammalian cells. Second, because patterns of protein interactions may be conserved throughout species, we searched for orthologs of known mammalian interactors and measured binary interaction with these predicted candidates. We found KIN-3 and KIN-10, the orthologs of CK2α and CK2β, as new partners of LET-756. Third, following the assumption that recognition motifs mediating protein interaction may be conserved between species, we screened a two-hybrid cDNA human library using LET-756 as bait. Among the few FGF partners detected was 14-3-3β. In support of this interaction we showed that the two 14-3-3β orthologous proteins, FTT-1 and FTT-2/PAR-5, interacted with LET-756.

**Conclusion:**

We have conducted the first extensive search for LET-756 interactors using a multi-directional approach and established the first interaction map of LET-756/FGF with other FGF binding proteins from other species. The interactors identified play various roles in developmental process or basic biochemical events such as ribosome biogenesis.

## Background

FGFs constitute a superfamily of pleiotropic growth factors involved in multiple cellular processes such as mitogenesis, angiogenesis and mesoderm induction [1]. There are 22 FGFs in humans. Except FGF11-14, they exert their biological activities by acting as extracellular growth factors binding to receptors (FGFR1-4) of the tyrosine kinase receptor superfamily [2]. In addition, FGF1-3 and FGF11-14 are localized in the nucleus and function intracellularly [3]. Intracellular FGFs bind to several proteins that play a role in FGF trafficking: FIBP, which allows FGF1 to shuttle between the cytosol and the nucleus [4], synaptotagmin-1, which allows FGF1 exocytosis [5], Cystein Rich FGF receptor (CFR), which forms complexes with various FGFs and allows their secretion [6,7], and LRP-1 and 2 (lipoprotein receptor-related proteins), which in conjunction with DAB-1 (Disabled) regulate EGL-17/FGF export in *C. elegans *[8]. FGF interactors may also regulate FGF nuclear activity; this is the case of Casein Kinase II regulatory subunits [9] and splicing factor SF3a66 [10], which both interact with FGF2. Finally, proteins of the extracellular matrix such as fibstatin and fibrinogen interact with FGF2 [11,12].

LET-756 is one of the two FGFs of *C. elegans *[13, 14 for reviews]. It is essential for worm development [15]. Like some mammalian FGFs, it acts both intra and extracellularly. The molecular motif allowing secretion [16] and some of LET-756 extracellular functions have been described [17,18] but the intracellular functions remain poorly defined, although nuclear localization is probably of importance [19]. To further characterize the functions of LET-756, we used yeast two-hybrid screens to identify proteins that interact with this FGF. We identified several interacting proteins involved in various developmental processes or in basic biochemical events such as ribosome biogenesis, and validated some of the interactions by co-immunoprecipitation and/or colocalization.

## Results

### Identification of nematode LET-756 binding proteins by yeast two-hybrid library screens

To identify worm proteins that interact with LET-756, we used the two-hybrid system in the MAV103 yeast with LET-756 fused to the Gal-4 DNA binding domain (pDB) as bait and two *C. elegans *libraries. The latter were either normalized [20] to contain one representative of each expressed gene of the whole genome (ORFeome) or derived from a mixed stage worm population. Library clones were coupled to the Gal-4 activating domain (pAD). The bait did not show any intrinsic transcriptional activation of the three yeast reporter genes. In a screen of approximately 4 × 10^6 ^transformants, 41 clones were positive for the two reporters tested, or for only one reporter but with great intensity. The gap repair technique confirmed 9 clones (Table 1). Sequencing of these clones and blastn or tblastx interrogation of databases revealed unidentified sequences (UIS) and sequences coding for proteins with known functions: UMP synthase, an enzyme involved in de novo nucleic acid synthesis, cathepsin (aspartic peptidase A1, pepsinogen family member), RPS-16 (small ribosomal subunit), transcription factor PAL-1 involved in the anterior-posterior development of the male [21], DAF-21, a chaperone of the HSP-90 family, involved in chemosensory transduction and insulin signalization [22,23], COL-129, an isoform of collagen, and SKR-2 (homolog of skp1 of *S. cerevisiae*), a component of the skp1p/cullin/F-box SCF complex with ubiquitin ligase activity [24,25]. The latter also shows similarities with P19, which is associated with cyclinA/CDK2 complex in humans.

To confirm the results on some on the interactors we judged potentially relevant, pAD interactor clones were picked individually from the ORFeome library and tested directly in yeast two-hybrid system against pDB LET-756. PAL-1 interacted strongly with LET-756 (two positive tests). For the other interactors, only one test was clearly positive (Table 2A).

### Identification of LET-756-binding proteins among orthologs of known mammalian FGF interactors

The conservation of signaling pathways between worms and mammals, as well as the conserved FGF structures in different species [26,27], suggested that orthologs of human FGF-binding proteins could interact with LET-756. We used blast interrogation to determine the most conserved orthologs supposed to retain the ancestral function of the known human FGF-interactors. These orthologs were recovered from the ORFeome library and tested in binary interactions (Table 2B). We found *kin-10 *(CK2β) and *rpl-6 *positive for two reporter genes, and *kin-3 *(CK2α), *hsp-6 *(mortalin), F14E5.2 (cystein rich FGF receptor, CFR) and C18A3.3 (NoBP) positive for one reporter gene.

### Identification of human LET-756-binding proteins

We based our third approach on the assumption that protein interaction motifs may be conserved between species. We made a heterologous screen using a cDNA human library as prey and LET-756 as bait. The yeast two-hybrid system was used with LET-756 fused to LexA DNA binding domain as bait (pDB) for the screening of a human placenta cDNA library containing the Gal-4 activating domain (pAD). Table 3 indicates the number of times the interactors were isolated and the strength of the interaction. Identified partners were different from those unveiled by the *C. elegans *screens but showed similar biologic activities (Table 4). MBD1 and ZN420 are transcription factors, 14-3-3β (YWHAB, tyrosine 3-monooxygenase/tryptophan 5-monooxygenase activation protein, β polypeptide) has chaperone activity, and FBLI1 (Filamin-binding LIM protein 1) is a protein of the Zyxin family.

Finally, the orthologs of human 14-3-3β *, ftt-1/par-5 *and *ftt-2*, and the orthologs of FBL1, *zyx-1a *and *zyx-1b*, were obtained from the nematode ORFeome library and tested for direct interaction in yeast two-hybrid system. Table 2C indicates that FTT-1 and FTT-2, but not ZYX-1a or ZYX-1b, reacted with the LET-756 bait.

### Confirmation of interactions by co- immunoprecipitation experiments

**Figure 1:**
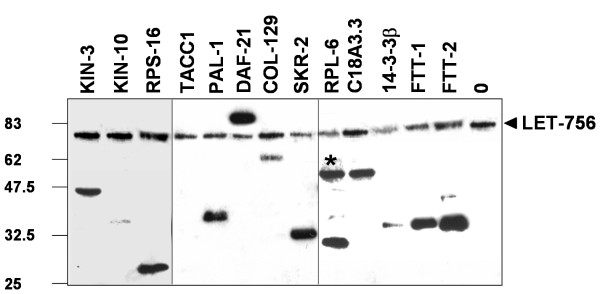
**Co-immunoprecipitation of LET-756::GFP and HA-tagged protein partners **Cos-1 cells were transfected with *let-756::gfp *alone (0) and with various HA-tagged constructs presumed or not (TACC1) to interact with LET-756. Twenty four hours later cell lysates were immunoprecipitated with anti-GFP antibodies. Western blots were first revealed with anti-HA antibodies and then with anti-GFP antibodies. An additional band, indicated with an asterisk, was consistently observed with HA- tagged RPL-6. A week and non- reproducible band can also be seen in the FTT-2 co-immunoprecipitation lane. None of these bands arose from possible complexes since 1) electrophoresis was performed in denaturating conditions and 2) antibody detection relied on the tag epitope and not on the endogenous protein.

A number of interactors we identified have been described as false positives in various studies. It is the case for HSP family members and ribosomal proteins, and to some extent for collagen-related proteins, Zn finger proteins and proteasome subunits [28]. To validate the interaction of LET-756 with the candidate partners, co-immunoprecipitation experiments were done in Cos-1 cells. Cos-1 cells were transiently cotransfected with HA-tagged partner constructs and LET-756::GFP, and the lysates were immunoprecipitated with anti-GFP. Fig. 1 shows the result of a western blot probed first with anti-HA to reveal the co-immunoprecipitated proteins, and second with anti-GFP to normalize the transfection with LET-756. All tested partners immunoprecipitated with LET-756, although with different strength, unrelated to their level of expression (not shown). The 14-3-3β and KIN-10 proteins were reproducibly the less efficient. To make sure that overexpression of the two tagged proteins was not responsible for the immunoprecipitation, we used TACC1 as an unrelated HA-tagged control. In similar condition, TACC1 was unable to immunoprecipitate LET-756.

### Confirmation of interactions by subcellular colocalization experiments

**Figure 2:**
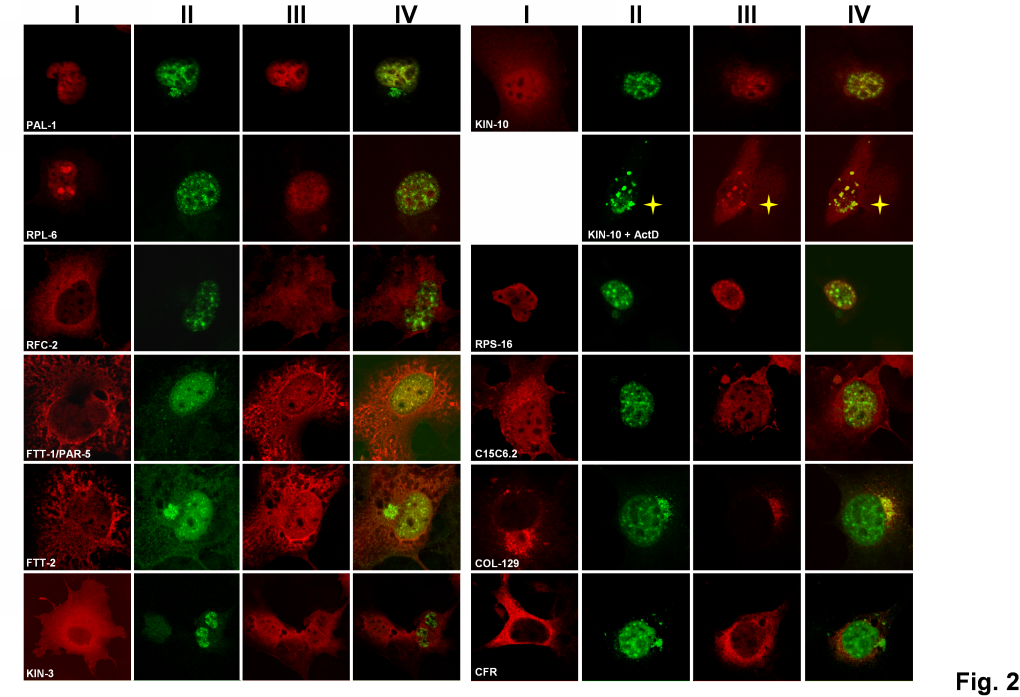
**Intracellular localization of LET-756::GFP and HA-tagged protein partners **Cos-1 cells were transfected with *let-756::gfp *with or without various HA-tagged encoded constructs. Twenty four hours later cells were either fixed in 4% paraformaldehyde and permeabilized in triton or in cold methanol. Cells were incubated for an hour with rat anti-HA antibodies. Secondary goat anti-rat Texas red-coupled antibodies were then added for another 30 min. Coverslips were visualized with a confocal Leica microscope. Cells were transfected with the HA-tagged protein alone (column I) or cotransfected with *let-756::gfp *and revealed by direct fluorescence (column II), by anti-HA immunofluorescence (column III) and merge (column IV). In cells cotransfected with LET-756 and KIN-10 and treated with actinomycin D, both GFP staining and anti-HA immunostaining are displaced around and into the nucleolus. The yellow star indicates a neighbor single KIN-10 transfected cells. Its localization is not modified by the drugs. Panels A and B each show results for five interactors analyzed.

To further confirm FGF/partner interaction in mammalian cells, HA-tagged partners were co-expressed with LET-756::GFP in Cos-1 cells and their respective subcellular localization was examined. As already described [16,19], LET-756::GFP localized in specific regions of the nucleus where splicing factors are concentrated. Immunofluorescence microscopy using anti-HA antibodies revealed PAL-1 in foci in the nucleus that colocalized with LET-756 (Fig. 2). We have previously established [19] that treatment of LET-756 expressing cells with actinomycin D (a drug inhibiting Pol I and Pol II activities) displaces LET-756 to the perinucleolar and nucleolar compartments. Addition of actinomycin D did not move the PAL-1 protein to the nucleolus as it did for LET-756 but kept both partners in close association in nucleoplasmic foci. Other partners, such as RPL-6, were delocalized by transfection of LET-756. RPL-6 was localized in large foci mainly in the nucleoplasm when transfected alone (column I, Fig. 2) but was most often dispersed through the nucleoplasm and associated with LET-756 when cotransfected with the FGF (Fig. 2, column II to IV). FTT-1/PAR-5 and FTT-2 localized preferentially in the cytoplasm when transfected alone, but observed also in the nucleus when transfected together with LET-756. In addition, vesicles containing both LET-756 and FTT-1 or FTT-2 were visible. KIN-3 colocalized with LET-756 in nuclei and exhibited a strong expression in cytoplasm whether LET-756 was present or not whereas KIN-10 present in nucleoplasm of untransfected LET-756 cells moved with LET-756 in the speckles when co-transfected. Upon actinomycin D treatment both LET-756 and KIN-10 formed enlarged speckles and moved to the nucleolus (Fig. 2). This stricking delocalization observed upon actinomycin D treated KIN-10 co-transfected cells did not occurred with KIN-3. In other instances, the partner modified LET-756 localization: RPS-16 concentrated LET-756 in large foci when both proteins were present in the nucleus (Fig. 2). The protein encoded by C15C6.2 did not show any gross colocalization (Fig. 2). Finally, COL-129 was localized only at the Golgi apparatus, whether LET-756 was present or not, and CFR was localized only in the cytoplasm.

## Discussion

Several growth factors are found in the nucleus in addition to their other localizations. This is the case for LET-756 but not for EGL-17, the other *C. elegans *FGF [29]. The role of LET-756 in the nucleus is not known. To help characterize this role we searched to identify intracellular binding partners of LET-756. By using different two-hybrid screens in yeast, we identified several proteins involved in various aspects of protein synthesis or degradation. Further analysis by co-immunoprecipitation and colocalization confirmed the interactions identified. We demonstrated that not only LET-756 could interact with mammalian partners as well as their orthologs (e.g 14-3-3β vs FTT-1/PAR-5 and FTT-2) but also that orthologs of mammalian FGF partners could interact with LET-756 (CK2β *vs *KIN-10). Analyses of these partners could be of interest in the study of mammalian FGFs.

The majority of the proteins we identified are nuclear, which was expected since the two-hybrid system needs the fusion to be targeted to the nucleus. However, some interactions have been identified as false positive in other screens [28]. By performing co-immunoprecipitations and studying subcellular localization, we validated the interaction of LET-756 with RPS-16, FTT-1/PAR-5, FTT-2, KIN-10 and RPL-6 with high score, and with PAL-1, DAF-21, SKR-2, KIN-3 and C18A3.3 with lower strength (see Table 6). The function of some interacting partners is relevant to FGF biology. The 14-3-3/FTT-1/FTT-2 proteins, which belong to the highly conserved family of chaperone molecules transit to the nucleus and participate in nucleo-cytoplasmic transport, regulating intracellular transduction [30]. We did not find a 14-3-3 conventional phosphorylated binding site on LET-756. However, other domains have been involved in 14-3-3 binding, such as nuclear localization signals (31 for review). No role for FTT-1 or FTT-2 in modulating secretion has been assigned in *C. elegans*. It will be interesting to analyze whether interaction of these proteins with the EFVSVA motif of secretion described in LET-756 [16] causes its secretion. The interaction of LET-756 with PAL-1 is of interest because PAL-1 is also highly conserved during evolution. It is the ortholog of caudal (*Drosophila*), CDX1, 2 and 4 (mammals) and Xcad3 (Xenopus) paraHOX proteins. Caudal proteins are involved in the transcriptional regulation of multiple genes that are involved in posterior patterning [32]. The interaction of LET-756 with PAL-1 could activate the expression of various genes involved in nematode anterior-posterior development as it is the case for the interaction of Xenopus e-FGF with Xcad3 and the resulting activation of *HOX *genes [33,34]. In addition, *pal-1 *mutant exhibits aberrant cell position in posterior muscle cells [35], a site of LET-756 expression [13,19] as well as in posterior hypodermis, a site of LET-756 action [17]. Both muscle and epidermis evolve from the C lineage. In the absence of PAL-1, the C blastomeres fail to develop. Protein phosphorylation by the coordinated activities of protein kinases and phosphatases is central to many signal transduction pathways. The combined action of LET-756/FGF, EGL-15 receptor, CLR-1 phosphatase (for a review see [14]) and KIN-3 and KIN-10, the respective catalytic and regulatory subunits of CK2, might regulate various processes involved in proliferation -as it is described for FGF1 and 2 [43,9] – or in other functions. KIN-3 and KIN-10 have been recently implicated in primary cilia biology [55]. Finally, some ribosomal proteins interact with mammalian FGF [36-38] to regulate their signaling and trafficking to the nucleus; reciprocally, FGFs may regulate ribosome biogenesis and protein synthesis during the G1 phase of the cell cycle. In contrast to these relevant interactors, others appear irrelevant, such as MBD1 since no methylation occurs in *C. elegans*.

The interactions revealed by the two-hybrid screens are rather weak. This could be due to 1) the high stringency associated with the system using the MAV 103/203 yeast strains; it is worth noting that in large-scale screenings of interactions no partner for LET-756 was found [20]; 2) a bad exposure of the binding site in the fusion proteins; 3) the existence of ternary interactions as seen in the ligand – tyrosine kinase receptor – heparan sulphate complex and 4) the need for post-translational modifications of the proteins that occur in mammalian cells and not in yeast, explaining why better interactions between glycosylated LET-756 [16] and various partners were obtained in immunoprecipitation and immunofluorescence experiments than in yeast two-hybrid screens.

Finally, it will be interesting to know whether the expression pattern of LET-756, which is mainly muscular and neuronal in the worm, overlap with that of the various partners. Search in the literature (56, 57) was not conclusive since the majority of the partners were found in eggs (FTT-1/PAR-5, FTT-2, DAF-21, PAL-1), intestine (HSP-6, SKR-2) or cuticle (COL-129).

## Conclusion

**Figure 3:**
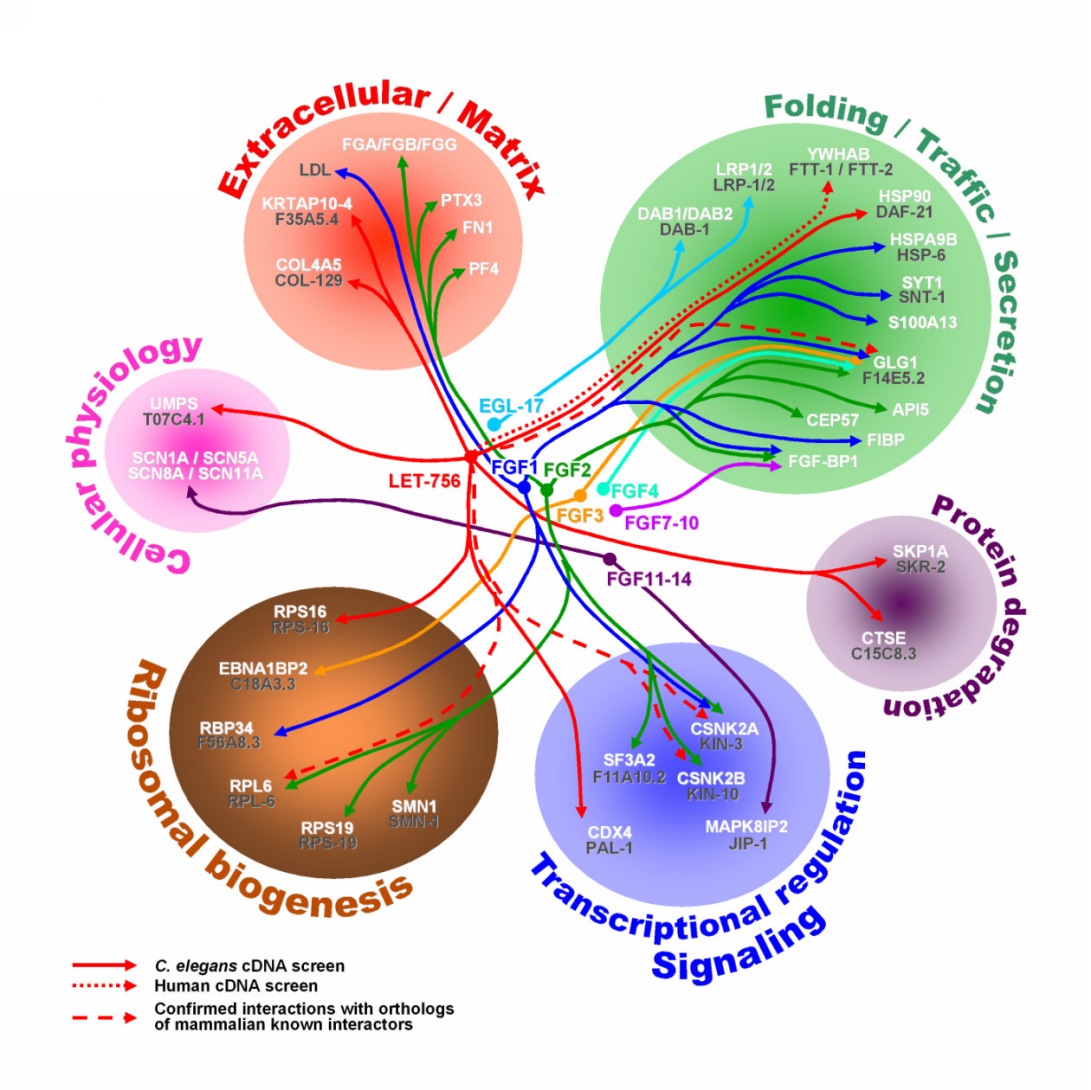
**Interactome of the FGF family **The identified interactors of various FGFs were grouped in six categories, depending of the functional activities of proteins. The human and *C. elegans *orthologs are indicated: human (white), worm (grey).

We have conducted the first extensive search for LET-756 interactors and established the first interaction map of LET-756/FGF with FGF binding proteins (Fig. 3 and Table 6). This could help understand FGF functions. Proteins of interest were involved in developmental processes or in basic biochemical events such as ribosome biogenesis and protein synthesis. In addition, to get insight in the evolution of the FGF interactome network, which we have illustrated in Fig. 3, we tested 6 of 20 orthologs of human FGF interactors (Table 6), and found KIN-10 and RPL-6 as new potential interactors. Looking for physical interactions in a physiological system will determine which of these interactions are essential.

In conclusion, 1) combining the yeast two-hybrid screen with bioinformatics and computational biology, we have delineated potential interactors of LET-756, and possibly of the entire FGF family; 2) comparative genomics analysis yielded valuable insights into conserved and divergent aspects of function, regulation, and evolution since not all pathways are conserved as demonstrated by the ortholog analysis; 3) the information given herein, although not complete, might be useful for people working in the field.

## Methods

### Yeast two-hybrid assays

A full-length *let-756 *transcript was fused in-frame with the coding sequence of the DNA binding domain (DB) of Gal4, and was used in a Y2H screen system as described in [20] for the *C elegans *libraries. Two worm libraries fused to the activation domain (AD) of Gal4, a cDNA and the AD-ORFeome libraries [53] were screened. The MAV103 yeast strain based Y2H assay contains three reporter genes (*HIS3*, *lacZ *and *URA3*) [54]. A cDNA human placenta library fused to the activation domain of LexA was also screened. The L40 yeast strain based Y2H assay contains only two reporters (*HIS3 *and *lacZ*). In this case, the full length *let-756 *transcript was cloned into the LexA DNA binding domain bait expression vector pBTM116B Kana. Yeast assays were done using conventional lithium acetate-based method. Clones were assigned scores for LacZ expression, growth on plates lacking histidine but containing 20 or 40 mM 3-amino triazol and in addition, growth on plates lacking uracil for the *C. elegans *libraries. To ascertain interactions, the gap repair technique was performed as in [54].

### Plasmid construction

To generate prey-tagged expression vectors used in co-immunoprecipitation assay or immunofluorescence, the coding regions of various genes were amplified by PCR using as template the corresponding EST clones obtained from RZPD (Berlin, Germany) and then inserted in the expression vectors using Gateway technology (Invitrogen, Carlsbad, CA).

LET-756::GFP was obtained as previously described [16].

### Cell culture and in vivo interaction assay

Cos-1 cells grown in DMEM supplemented with 10% fetal calf serum were plated in 60-mm dishes at a concentration of 2 × 10^6 ^cells/dish and immediately transfected with 1μg DNA in Fugene, according to the manufacturer instructions. Twenty four hours after transfection, cells were lysed in 1 ml triton buffer (10 mM Tris, pH7.4, 100 mM NaCl, 2.5 mM MgCl2, 1% triton, 1 mM EDTA, 10 mM DTT). Detergent insoluble materials were removed by 30 min centrifugation at 13000 rpm at 4°C. Whole cell lysates were first incubated with protein G-sepharose beads and then with the relevant antibody for at least 2 hr. Protein G-sepharose beads were then added for another additional 2 hr and washed 3 times with lysis buffer. Bound proteins were eluted by boiling in SDS sample buffer and resolved on a 10% SDS-PAGE gel and analyzed by Western blots. For immunofluorescence analysis, cells grown on glass coverslips were fixed and permeabilized in 3.7% PAF and 0.1%Triton or in methanol for 6 min at -20°C. Similar results were obtained using these different modes of fixation. Cells were incubated with primary antibody for 1 hr and then incubated with Texas Red-conjugated secondary antibody for another hr. Plasmid LET-756::GFP was visualized by autofluorescence. Coverslips were examined using a Leica TCS NT confocal microscope.

The following antibodies were used: rat monoclonal anti-HA (12CA5) antibody was from Roche (Indianopolis, IN, USA), rabbit polyclonal anti-GFP from Abcam (Cambridge, UK), Texas Red anti-rat antibody from Molecular Probes (Eugene, OR, USA), peroxydase anti-mouse from Santa Cruz (Santa Cruz, CA, USA)

## Abbreviations

DMEM, Dulbecco's mofied Eagle's medium; FBS, fetal bovine serum; DTT dithiotreitol; Y2H, yeast two hybrid; GFP, green fluorescent protein

## Authors' contributions

CP, YB and AH performed the two-hybrid screenings and the analyses of the data, CP and FC the gap-repair confirmation of clones, FC and RR the co-immunoprecipitation and immunofluorescence experiments, CP the art work. CP played the major role in the bioinformatics analysis. DB initiated the *C. elegans *project and helped draft the manuscript. RR conceived and coordinated the study and wrote the manuscript. All authors read and approved the final manuscript.

## Figures and Tables

**Table 1:** LET-756-interacting proteins identified by screening Y2H *C. elegans *libraries. Bait used was DB-LET-756. Sequences of AD-interactor clones are in (58). Clones were assigned scores for LacZ expression, growth on plates lacking histidine but containing 20 or 40 mM 3-amino triazol (3-AT) and in addition, growth on plates lacking uracil (URA) for the *C. elegans *libraries

**Interactor **(sequence name)	Gene name	**Human ortholog(s)**	**LacZ**	**3-AT 20 mM**	**3-AT 40 mM**	**URA**	**Library**
AD-C38D4.6	*pal-1*	CDX4 (Caudal-type homeobox protein 4)	++	+	-	-	cDNA
AD-C47E8.5	*daf-21*	HSP90 family	-	++	+	-	cDNA
AD-T01C3.6	*rps-16*	RPS16 (40S ribosomal protein S16)	+/-	++	-	-	cDNA
AD-T07C4.1	-	UMPS (UMP-synthase)	-	+/-	-	++	cDNA
AD-C15C8.3	-	CTSE (Cathepsin E precursor)	+/-	+	-	-	cDNA
AD-C15C6.2b	C15C6.2	-	-	+	+	-	cDNA
AD-M18.1	*col-129*	COL4A5 (Collagen alpha 5(IV) chain precursor)	+/-	+	+/-	-	cDNA
AD-F35A5.4	-	KRTAP10-4 (Keratin-associated protein 10-4)	+	-	-	-	AD-ORFeome
AD-F46A9.4	*skr-2*	SKP1A (S-phase kinase-associated protein 1A)	+/-	++	-	-	AD-ORFeome

**Table 2:** Direct interactions between LET-756 (DB) and clones derived from the ORFeome library. A) clones detected in the screening of the *C. elegans *libraries; B) orthologs of clones detected in the screening of the human placenta library; C) orthologs of known mammalian FGF interactors.

	**Interactors (AD)**	**LET-756 (DB)**		
		**LacZ**	**3-AT**	**URA**

A	RPS-16	**+/-**	**++**	**-**
	PAL-1	**++**	**+**	**-**
	DAF-21	**-**	+*	**-**
	COL-129	**+/-**	**+**	**-**
	SKR-2	**+/-**	**+/-**	**-**
	C15C6.2	**+/-**	**+**	**-**

**B**	KIN-10 (CSNK2B, CK2β)	**+/-**	**+**	**-**
	KIN-3 (CSNK2A, CK2α)	**-**	**+**	**-**
	HSP-6 (HSPA9B, mortalin)	**-**	**+**	**-**
	F14E5.2 (GLG1, CFR)	**-**	**+/-**	**-**
	C18A3.3 (EBNA1BP2, NoBP)	**-**	**+/-**	**-**
	RPL-6 (RPL6)	**+/-**	**+**	**-**

**C**	FTT-1/PAR-5 (YWHAB, 14-3-3β)	**+/-**	**+**	**-**
	FTT-2 (YWHAB, 14-3-3β)	**+/-**	**+**	**-**
	ZYX-1a, ZYX-1b (ZN420)	**-**	**-**	**-**

*: 3-AT concentration allowing yeast growth was 20mM except for DAF-21 where it was 40 mM

**Table 3A:** LET-756-interacting proteins identified by screening Y2H human placenta libraries. A: Number of times the clone was detected during the Y2H library screening and scores for the different reporter genes.

**Interactor**	**times**	**LET-756**			
		lacZ	His-3	3-AT (20 mM)	3-AT (40 mM)
YWHAB (14-3-3 β)	11	+	+	-	-
MBD1	1	+	+	-	-
EIF3S2 (eiF3)	1	+	+	-	-
FBLP1	1	+	+	-	-
KRAB100	3	+	+	+	+

*: 3-AT concentration allowing yeast growth was 20mM except for DAF-21 where it was 40 mM

**Table 3B:** LET-756-interacting proteins identified by screening Y2H human placenta libraries. B: Description of the proteins and identification of the *C. elegans *ortologs.

**Protein**	**Description**	***C.elegans *orthologs**
YWHAB (14-3-3β)	protein with chaperone activity implicated in subcellular compartmentalization of binding partners	PAR-5 or FTT1, FTT-2
MBD1	negative regulator of transcription, the methyl CpG-Binding Domain	none
EIF3S2	initiator of translation (subunit 9)	EIF3-B
FBLI1	protein belonging to the Zyxin family, having 3 LIM domains, involved in protein-protein interaction (filamin-binding LIM protein-1)	ZYX-1
ZN420	zinc finger protein with a Krab domain	none

*: 3-AT concentration allowing yeast growth was 20mM except for DAF-21 where it was 40 mM

**Table 4:** Summary of interaction strengths

**Interactors**	**Y2H- screen**	**Co-IP**	**Coloc**	**Deloc**	**Class**
RPS-16	++	++	+	+	I
FTT-1	++	+++	-	+	I
FTT-2	++	+++	-	+	I
KIN-10	++	+	+	+	I
PAL-1	+++	+	+	+	I
C15C6.2	++	++	+	-	II
RPL-6	++	++	-	+	II
DAF-21	+	++	ND		III
COL-129	++	+	+	-	III
SKR-2	++	++	ND		III
KIN-3	+	++	+/-	-	III
C18A3.3	+	++	ND		III

Scores obtained in the different assays used: (i) two-hybrid assays, (ii) co-immunoprecipitation (co-IP), (iii) colocalization by immunofluorescence in single transfected cells (Coloc) and (iv) delocalization (Deloc) of one or the two partners induced by the cotranfection. Interactions were classified as I (the strongest) to III (the weakest).

**Table 5:** *C. elegans *orthologous genes of known human FGF interactors.

**FGF**	**Interactor**	***C. elegans *or human orthologs**
FGF1	HSPA9B (Stress-70 protein, mortalin) [39]	*hsp-6 *(C37H5.8)
FGF1	NP_060979.2 (PRO1855, LOC55379, ribosome-binding protein p34, RBP34*) [39]	F56A8.3
FGF1	LDL (Low density lipoproteins) [40]	-
FGF1	S100A13 (Calcium binding protein S100A13) [41]	-
FGF1	SYT1 (Synaptotagmin-1) [5]	*snt-1 *(F31E8.2)
FGF1	FIBP (FGF-1 intracellular binding protein) [4]	-
FGF1 and FGF2	FGFBP1 (Fibroblast growth factor binding protein 1) [42]	-
FGF1 and FGF2	CSNK2A (Casein Kinase II, catalytic subunit α) [43]	*kin-3 (*B0205.7)
FGF2	CSNK2B (Casein Kinase II, regulatory subunit β) [9]	*kin-10 *(T01G9.6b)
FGF2	RPL6 (Large ribosomal subunit L6) [36]	*rpl-6 *(R151.3)
FGF2	RPS19 (40S ribosomal protein S19) [37]	*rps-19 *(T05F1.3)
FGF2	CEP57 (Translokin, Centrosomal protein of 57 kDa, Proliferation-inducing protein 8, PIG8) [44]	-
FGF2	SMN1 (Survival motor neuron protein) [45, 46]	*smn-1 *(C41G7.1)
FGF2	FGA/FGB/FGG (Fibrinogen, alpha/beta/gamma polypeptides) [12, 47]	-
FGF2	FN1 (Fibstatin, fragment of fibronectin) [11]	C56C10.4
FGF2	API5 (Apoptosis inhibitor 5, Fibroblast growth factor 2-interacting factor, FIF) [48]	-
FGF2	SF3A2 (Splicing factor 3A subunit 2) [10]	F11A10.2
FGF2	PTX3 (Long pentraxin 3) [49]	-
FGF2	PF4 (CXC chemokine platelet factor 4) [50]	-
FGF1, FGF2, FGF3, FGF4	GLG1 (Golgi apparatus protein 1 precursor, Cysteine-rich fibroblast growth factor receptor, CFR-1) [6,7]	F14E5.2
FGF3	EBNA1BP2 (rRNA processing protein EBP2, Nucleolar Binding Protein, NoBP) [51]	C18A3.3
FGF11-14	MAPK8IP2 (Islet-brain-2, JNK MAP kinase scaffold protein 2) [52]	*jip-1 *(F56D12.4)
EGL-17	LRP-1 (Low-density lipoprotein Receptor Related, F29D11.1) [8]	*lrp-1*
EGL-17	LRP-2 (Low-density lipoprotein Receptor Related, T21E3.3) [8]	*lrp-2*
EGL-17	DAB-1 (Drosophila disabled homolog, M110.5) [8]	*dab-1/dab-2*

- No approved gene name
